# Evaluating visual and auditory contributions to the cognitive restoration effect

**DOI:** 10.3389/fpsyg.2014.00548

**Published:** 2014-06-05

**Authors:** Adam G. Emfield, Mark B. Neider

**Affiliations:** Applied Cognition and Aging Lab, Department of Psychology, University of Central FloridaOrlando, FL, USA

**Keywords:** cognitive restoration, Attention Restoration Theory, natural environments, urban environments, immersion, mood, attention

## Abstract

It has been suggested that certain real-world environments can have a restorative effect on an individual, as expressed in changes in cognitive performance and mood. Much of this research builds on Attention Restoration Theory (ART), which suggests that environments that have certain characteristics induce cognitive restoration via variations in attentional demands. Specifically, natural environments that require little top-down processing have a positive effect on cognitive performance, while city-like environments show no effect. We characterized the cognitive restoration effect further by examining (1) whether natural visual stimuli, such as blue spaces, were more likely to provide a restorative effect over urban visual stimuli, (2) if increasing immersion with environment-related sound produces a similar or superior effect, (3) if this effect extends to other cognitive tasks, such as the functional field of view (FFOV), and (4) if we could better understand this effect by providing controls beyond previous works. We had 202 participants complete a cognitive task battery, consisting of a reverse digit span task, the attention network task, and the FFOV task prior to and immediately after a restoration period. In the restoration period, participants were assigned to one of seven conditions in which they listened to natural or urban sounds, watched images of natural or urban environments, or a combination of both. Additionally, some participants were in a control group with exposure to neither picture nor sound. While we found some indication of practice effects, there were no differential effects of restoration observed in any of our cognitive tasks, regardless of condition. We did, however, find evidence that our nature images and sounds were more relaxing than their urban counterparts. Overall, our findings suggest that acute exposure to relaxing pictorial and auditory stimulus is insufficient to induce improvements in cognitive performance.

## Introduction

An increasing number of people have chosen to call urban areas their homes. In the US, it is estimated that 82% of the population resides in cities and suburbs. World-wide, the urban population is approximately 50.5%, with an annual increase of 1.85% (Urbanization, 2011). Despite our continued urbanization, many people spend a fair amount of their time and money trying to *leave* urban environments in favor of recreational activities in locales where nature is more prevalent than concrete (e.g., taking a hike in the woods or spending a day at the beach). Given the trend that people migrate toward urban areas when establishing residence, what is it that draws them back out to nature, and are there tangible benefits associated with natural environments?

The desire to spend time in natural environments has been discussed extensively, and considered heavily in the context of urban planning (Olmsted, 1870). A growing number of studies seem to support increased access to nature-like environments in urban settings (e.g., parks). There also appears to be an emerging consensus that spending time in natural environments engenders tangible benefits in both self-reported well-being (Ulrich et al., 1991; Herzog et al., 1997; Staats and Hartig, 2004; Hartig and Staats, 2006) and overall cognitive function (Hartig et al., 2003; Berman et al., 2008). Furthermore, it has been suggested that exposure to pictures of natural environments may reduce pain in patients undergoing bone marrow aspiration and biopsies (Lechtzin et al., 2010), and can result in many other health and wellness benefits (Velarde et al., 2007; Depledge et al., 2011).

The mechanisms underlying these nature-related benefits, however, have been the subject of much debate, and several theories have emerged to provide a framework within which existing findings might be contextualized. Two of these theories are Stress Reduction Theory (SRT; Ulrich, 1983) and Attention Restoration Theory (Kaplan and Kaplan, 1989; Kaplan, 1995). SRT focuses primarily on the effects of natural environments on affect, and suggests that spending time in natural environments evokes a positive initial affective response and concomitant change in physiological responses indicative of stress reduction. Indeed, numerous studies have provided some support for this by demonstrating an increase in positive affect and a drop in blood pressure (Hartig et al., 2003), reduced heart rate (Laumann et al., 2003), and a series of other physiological stress measures (Ulrich et al., 1991; van den Berg and Custers, 2011) when observers are exposed to natural environments. However, the benefits of exposure to natural environments are not limited to improvements in affect and reductions in physiological stress; changes in cognitive function may also reflect benefits.

Here we focus on ART, which credits improvements in cognitive performance to the restoration of direct attention after fatigue (Kaplan, 1995). Specifically, ART argues that the act of directing attention requires effort and leads to fatigue, reducing a person's ability to maintain performance, remain vigilant, and even increases the likelihood of irritability. One key element of this fatigue is a reduced ability to inhibit distraction. Importantly, once attentional capacity begins to diminish, it may be restored through various means, including traditional rest and sleep. However, interesting questions also arise when this is examined in the short-term; ART argues that, under the correct circumstances, the restoration of direct attention can take place by being exposed to the correct type of environment. In order to overcome fatigue, this environment must have four elements: (1) it must *be away* from the fatigue-inducing environment and from the typical daily environment of a person; (2) it must *have extent*, or be coherent, connected, and extensive enough to provide sufficient richness to captivate the mind; (3) it must *cause soft fascination*, or effortlessly hold the individual's attention; and (4) it must *be compatible* with the person's task-at-hand, such that it allows for restoration without distraction. Per ART, an environment which contains all four of these elements, in sufficient quantity, should be restorative.

Traditionally, the literature has discussed nature as being a fine exemplar of a restorative environment. In particular, the aquatic environments used in the current study meet the four requirements for restorativeness (Kaplan, 1995). Oceans and beaches meet the criteria of being away conceptually. These spaces are also coherent and rich, and can engage the mind, giving them extent. They also cause soft fascination, or involuntary attention capture, through a moderate level of stimulation that requires limited need for thought. Finally, a person who seeks such an environment as a method of restoration and reflection will find compatibility, with little to distract from these goals. The theory also argues that most urban environments are sufficiently lacking in one or more of these areas (Herzog et al., 1997), though some urban environments, such as museums, may still fit the bill for restoration (Kaplan et al., 1993).

The criteria for elucidating soft fascination warrants further discussion in the context of cognitive restoration. More recently, research has begun to discuss ART in the context of the different cognitive processes that attention may require (Berman et al., 2008). The involuntary attention capture in nature primarily requires bottom-up processing, which is sufficient to hold attention, but in a limited manner. Conversely, an urban environment results in more dramatic attention capture and a greater level of directed attention.

Research on the benefits of cognitive restoration has focused on the observation of changes in task performance after exposure to natural or urban environments. When exposed to a natural environment, directly or through pictures, research has shown improved performance on the Necker Cube Pattern Control Task (Tennessen and Cimprich, 1995; Taylor et al., 2002; Hartig et al., 2003), the digit span task (Tennessen and Cimprich, 1995; Taylor et al., 2002; Berman et al., 2008), and the Attention Network Task (ANT; Berman et al., 2008). For example, Berman et al. (2008) demonstrated that the number of correct trials on a digit span task increased three times as much after walking in a natural environment compared to walking in an urban setting, and increased by nearly 30% after simply viewing pictures of Nova Scotia when compared to exposure to urban pictures. Likewise, performance on the executive control portion of the ANT improved when participants were exposed to nature pictures, and decreased when participants were exposed to urban pictures. A similar pattern has been observed on the backward digit span task in individuals with depression (Berman et al., 2012) and in children with attention deficits (Taylor and Kuo, 2009).

However, despite previous studies, questions regarding the robustness of the cognitive restoration effect remain open. White et al. (2010) raised concerns about the images in previous studies lacking standardization; in some cases, there were people in the nature images and natural elements such as trees and water in the urban scenes. In fact, this study indicated that blue spaces and coastal regions (White et al., 2013) are perceived as particularly restorative, and are perceived as more restorative than primarily “green” nature scenes. Thus, we focused on aquatic environments containing some vegetation in the present study. Further, the presence of people in these environments has a certain social element, which may influence the results as well (Staats and Hartig, 2004). For example, a natural scene that could potentially be dangerous may appear safer when another person is present. This is an even larger concern in studies where participants walk in real-world environments, as control over the environment is limited at best. Additionally, in some cases significance was found on a given cognitive task, such as the search and memory test (Hartig et al., 1996) in one experiment, but was not replicated in other experiments (Hartig et al., 2003). In a similar study that used the ANT (Larsen, 2011), no restorative effects on cognitive performance were detected, despite an effort to address many of the previous concerns. Indeed, a meta-analysis comparing the results in many of these studies found that, while nature shows beneficial effects on cognitive performance, these effects are no longer significant when adjustments are made for pre-test differences (Bowler et al., 2010).

The strength of the cognitive restoration effect has also been recently called into question. Specifically, when using pictures alone, restorative effects are often attenuated when compared to walking in the environments. In fact, in many cases, follow-up studies intended to characterize the cognitive restoration effect have reverted to using an actual walk in nature in lieu of pictures-based exposure (e.g., Berman et al., 2012 compared to Berman et al., 2008). The trade-off between ecological validity and experimental control becomes particularly difficult to manage in fully realistic environments (e.g., walking through an urban area), and thus far little has been done to try to balance both. Immersive environments might provide one way to reconcile ecological validity with experimental control. In the domain of virtual reality, much research has been done to investigate how to make a person feel more present, or immersed, in a virtual environment (Stone, 2008). In this context, immersion can be loosely equated to the realism of an environment (Brown and Cairns, 2004). While some have proposed the creation of life-like virtual reality and the use of CAVEs to further control studies evaluating ART (Depledge et al., 2011), there may be simpler ways to increase immersion in the laboratory without the risks of virtual reality, such as motion sickness and nausea. For example, the simple addition of sound has been shown to increase immersion in video games (Grimshaw, 2008; Grimshaw et al., 2008) and in virtual reality (Serafin and Serafin, 2004; Sanders and Cairns, 2010). Additionally, nature sounds, such as birdsong, are perceived to be restorative (Ratcliffe et al., 2013). In the context of ART, the addition of sound should help increase the level of *extent* found in a natural environment.

The current study had four primary goals. First, we aimed to replicate the previous work of Berman et al. (2008) using a similar experimental design and set of tasks to investigate the effects of natural environments on both cognitive performance and mood. Second, we attempted to increase the level of environmental immersion by including environmentally consistent sounds, with the supplementary aim of increasing ecological validity above that of pictures alone, while maintaining experimental control above that of walking in the environments. Additionally, this allowed us to extend the current literature by investigating the effect that nature and urban sounds alone have on restoration. Third, we extended our research to include another cognitive task—the functional field of view (FFOV; Mackworth, 1965; Engel, 1971, 1977; Bouma, 1978; Ball et al., 1988) test. This test assesses the breadth of attentional distribution from the point of regard and is thought to indicate from which portions of the field of view useful information can be extracted. Performance on this task has been shown to be related to performance on driving tasks, which also require directed attention (e.g., Crundall et al., 1999; Roenker et al., 2003; Atchley and Dressel, 2004) and has been shown to be malleable through training (Ball et al., 2007; Belchior et al., 2013). It stands to reason that if a person has a greater ability to use directed attention (as they might after cognitive restoration), the person should perform this task more quickly after restoration. Finally, we included a control group that did not get access to any of the environments, against which we could compare environmentally induced changes in cognitive performance. Previous studies have neglected to include a control group (e.g., Berman et al., 2008, 2012), and by including one here we accounted for some of the concerns raised by others (White et al., 2010; Depledge et al., 2011).

To accomplish these goals, we had participants complete a battery of cognitive tasks, including several that have been previously found to be sensitive to cognitive restoration (e.g., Tennessen and Cimprich, 1995; Berman et al., 2008, 2012; Taylor and Kuo, 2009), and the additional FFOV. Participants also rated current affect using the Positive and Negative Affect Scale (PANAS; Watson and Clark, 1988). After completing the cognitive battery and affect measure, participants were exposed to pictures (urban or nature), sounds (urban or nature), or a combination of both for a set amount of time (participants subjectively rated how relaxing the stimuli were during exposure). After viewing/listening to the picture/auditory stimuli, participants completed the cognitive test battery a second time. Changes in performance on the cognitive battery between the first and second administration provided evidence for or against the cognitive restoration effect.

Our predictions were largely consistent with the previous literature on ART and SRT. We expected that participants would rate the nature images and sounds as more relaxing than urban images and sounds. We also predicted that participants in the nature conditions would exhibit a larger increase in positive affect than control and urban conditions, with a greater decrease in negative affect (as measured by the PANAS). Additionally, we predicted that those in the nature conditions would experience greater improvements in cognitive performance on the cognitive task battery when compared to control and urban conditions. Finally, we expected that when both pictures and sounds were combined in either type of environment, the resulting effect (benefit for nature scenes and cost for urban scenes) would be stronger than when visual or auditory stimuli of either environment type were presented in isolation.

## Methods

### Participants

Participants were 202 undergraduate students (128 female; age *M* = 19.8) recruited from the University of Central Florida's psychology department subject pool, and were compensated with course credit. Research was approved by the university's Institutional Review Board, and all participants provided informed consent. Vision was tested using a Snellen chart, with all participants scoring 20/25 vision or better. Color blindness was evaluated using the Ishihara color plates 1–13; any participants displaying any color vision impairment were excluded. Ten participants were excluded (1 female); two due to technical difficulties, four due to color blindness, three for failing to follow the experimental protocol, and one voluntarily withdrew.

Demographic information was collected from all participants and included age, gender, race, handedness, and level of education. In addition, we asked about the location in which participants lived for the longest period of time: Urban, Suburban, or Rural, with participants predominantly from suburban areas (124), and an equal number from rural and urban areas (34 each). Participants reported their current waking state and arousal level using the Stanford Sleepiness Scale (Hoddes et al., 1973).

### Design

We employed a between-subjects design in our study. Participants were randomly assigned to one of seven conditions, where numbers in parentheses indicate number of participants per condition: Control (27), Nature Sounds (28), Nature Pictures (27), Nature Both (28), Urban Sounds (28), Urban Pictures (27), or Urban Both (27).

### Apparatus

Data were collected using two Dell Inspiron 570 computers, each with a Dell P190S 19″ flat panel LCD monitor. Participants were seated 65 cm from the monitor, and wore Audio-Technica ATH-ANC7B noise-cancelling headphones throughout the experiment.

### Materials

**Figure 1:**
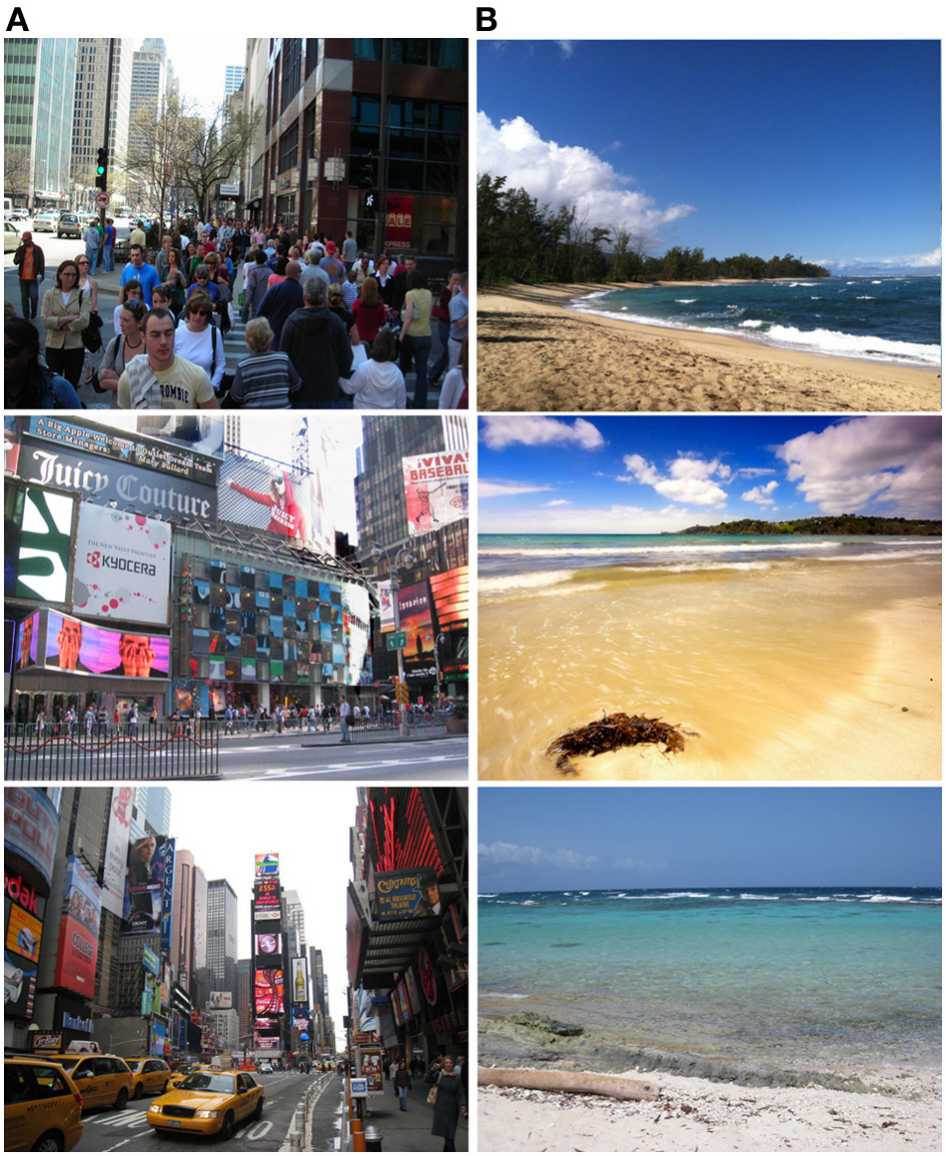
**Sample images from the experiment**. **(A)** Images are from the urban set, while **(B)** are from the nature set.

The image sets were created from a large number of pictures collected from various sources on the internet, as well as from some personal collections. From the large set, we selected 50 images for nature and 50 for urban environments that best fit our criteria for a controlled image set. For both sets, images were taken at approximately eye height, were high resolution, and could be fit to the screen without any skew or blur. All images were taken with good lighting during the daytime, in good weather, with consistency in lighting and weather across images. The resolution of all images met or exceeded the resolution of the display, preventing any blurring or stretching of images, which was one a concern noted in a previous study on the topic (Larsen, 2011). Additionally, images were required to be subjectively coherent with each other, such that no image stood out from the rest in the set. Sample images for each image set (natural and urban) are displayed in Figure 1.

For the nature images, we chose to use images of the beach and ocean. None of the images were composed entirely of water or of beach, with some containing trees and grass, nor did they contain any people or man-made objects or structures, in an attempt to reduce the need for top-down processing and to limit the presence of elements that might co-occur in urban environments. In order to limit the likelihood of causing a stress or negative affect response, we avoided images with features such as large waves or large cliffs.

The urban images were all of large, major cities in the United States or in Europe. Images were required to have people present and be primarily composed of man-made structures, consistent with most modern urban environments. Additionally, urban scenes typically contained some automobile traffic. Efforts were made to ensure urban images contained as little “nature” (e.g., trees, water, etc.) as possible, and no image had any major natural features. If any signs were present, they were in English.

For environmental sounds, we chose sounds that were as consistent as possible with their respective picture conditions, with the intention of increasing immersion and minimizing dissonance of the conditions with both images and sound. For the nature conditions, we used a single audio track of gentle waves lapping on the beach, with some occasional sounds of a light breeze or of seagulls (Joseph, 2010). For the urban conditions, we used an audio track recorded in Times Square in New York City (Times Square, 2000). This included the sounds of people talking, cars driving and honking, and other background noises from the city.

### Measures

Mood was evaluated using the PANAS. The PANAS is a test that has participants rate their mood at “the current moment” when presented with a word, such as “excited.” All ratings are given on a 5-point Likert scale, where 1 = very slightly or not at all, and 5 = extremely. On the PANAS, a higher score indicates a higher level of affect in a given direction; that is, a high score on the negative component indicates a greater level of negative affect. Scores range between 10 and 50 for each type of affect. Word order was determined randomly for each participant. Ratings are divided into two categories, resulting in a score for positive affect and another for negative affect. It has been used in previous studies investigating ART (e.g., Berman et al., 2008, 2012), though findings related to the PANAS have not always been consistent across studies.

Cognitive performance was evaluated using a task battery consisting of the backward digit span task (Cowan, 2001), the ANT (Fan et al., 2002), and a FFOV task. Each of these tasks, as well as the PANAS, was presented using the E-Prime 2.0 software (Psychology Software Tools, Pittsburgh, PA).

Images and sounds were rated for how relaxing the participants found them, using a 7-point Likert scale. In picture and both conditions, all images were rated, and in sound and both conditions, the sound file was rated. A score of “1” represented the “least relaxing,” and a score of “7” was the “most relaxing.”

#### Backward digit span task

The backward digit span task is one of the most frequently used tasks in studies investigating ART, and one with some of the most consistent findings. Participants were presented with a series of numbers 3–9 digits long through noise-canceling headphones. After the series of numbers finished playing, participants entered the digits in reverse order using the keyboard. Any time a participant completed two trials correctly the length of the subsequent string increased by one digit. If either trial contained a mistake, the string length decreased by one digit. There were a total of 14 trials, resulting in a maximum string length of nine digits. The score was determined by the last string length in which two trials were correct, indicating the participant's digit span capacity.

It is worth noting that in scoring the backward digit-span task, some previous studies have used number of correct trials as a score (e.g., Berman et al., 2008); however, our scores represent digit span *capacity*, not the number of correct trials. For example, two different participants with a digit span capacity of 7 can have a different number of correct trials; the first participant could have 10 correct trials in a row, and then miss the final four trials, while the second could also get 10 correct in a row, and then make a mistake on every other one for the remaining four, resulting in 12 correct trials.

#### ANT task

The ANT (Fan et al., 2002, 2005) task is a combination of the Posner spatial cueing task (Posner, 1980) and the Ericksen flanker task (Eriksen and Eriksen, 1971), and was obtained from the Sackler Institute website (http://www.sacklerinstitute.org). The script was originally written for an older version of E-Prime, but was converted to version 2.0 for the current study. It differentiates between alerting, orienting, and executive attentional functions through the use of two different types of cues—spatial and temporal. In this task, spatial cues indicate the location or the orientation of the target stimulus, while the temporal cues alert the participant to the approximate onset of the target. Each trial was composed of a fixation, followed by a cue, and the presentation of the target and its flankers. The target was “flanked” by two arrows on each side, which either pointed in the same direction (congruent) or in the opposite direction (incongruent) as the target. Additionally, flankers could be neutral, with dashes flanking the target. The participant's task was to indicate which direction the central arrow in an array of five arrows was pointing using the left or right button on a mouse, which was held in both hands. For our experiment, participants completed 24 practice trials, followed by 144 experimental trials (48 neutral, 48 congruent, 48 incongruent for the flankers; 36 spatial-cue, 36 central-cue, 36 no-cue, and 36 double-cue).

#### Functional field of view task

In the FFOV task, each trial began with a central fixation square superimposed over a black background for 1.25 s. Next, a screen appeared with 24 distractor squares distributed symmetrically at 10, 20, and 30° on eight different lines, or “spokes.” Between two of the spokes, one target triangle enclosed in a circle appeared at 10, 20, and 30° for 25, 50, or 75 ms during practice, and 10 ms during experimental trials. Once the target disappeared, participants viewed a screen with the eight spokes, and were instructed to click the spoke located in the same location as the target stimulus (regardless of distance from the center). Participants received accuracy feedback after each trial.

### Procedure

After providing informed consent, participants were screened for suitability for the study, and then seated in front of the experimental computer. They completed the demographic form and a PANAS to establish baseline affect. After the PANAS, participants completed the first cognitive test battery (pre-test), with the order of the tasks determined at random. Participants were not given a break between tasks, and quickly moved from one task to the next, in order to facilitate fatigue of directed attention. Approximate duration of the task battery was 30 min.

After completing the pre-test for the cognitive battery participants were exposed to the assigned treatment or control condition (restoration period). For the sound only conditions, participants listened to the appropriate sound (nature or urban) for 350 s while looking at a neutral gray screen, and selected the relaxation rating at the end of the restoration period. For the picture only conditions, participants viewed 50 images (nature or urban) for 7 s each, rating the how relaxing the image was after each image. For combined conditions, participants experienced both pictures and sounds, rated each image, and rated the sound at the end of the restoration period. For the control condition, participants viewed a neutral gray screen without sound for 7 min. The entire restoration period (exposure to pictures, sounds, or nothing) lasted approximately 7–10 min, depending on the speed at which participants rated images (actual exposure to stimuli was identical across all participants).

After the restoration period, the participants immediately completed the PANAS, followed by the post-test battery, which was identical to the pre-test. Tests were randomized independently of the pre-test order. Participants were then debriefed. The entire experiment lasted for approximately 90 min.

### Statistical analyses

Unless otherwise noted, analyses were conducted using two-way mixed ANOVAs, with time (pre-test, post-test) as the repeated-measure variable and the type of environment (condition; nature sounds, nature pictures, nature both, urban sounds, urban pictures, urban both, or control) as the between-subject variable. Any main effects of time indicate practice effects, regardless of condition; however, it is the interaction between time and condition that is of the most relevance to our predictions; a significant interaction would indicate that the exposure to an environment resulted in a differential change over time on performance on the cognitive tasks.

Additionally, to better characterize support for null effects in our data set, we also report posterior probabilities [*p*_BIC_(H_1_ | D)], which provide a graded probability indicating which hypothesis (the null or alternative) is better supported by the data. A *p*_BIC_(H_1_ | D) >0.5 indicates support for the alternative hypothesis, whereas a *p*_BIC_(H_1_ | D) < 0.5 indicates support for the null hypothesis (Masson, 2011). All reported *p*_BIC_(H_1_ | D) values are rounded to the second decimal.

## Results

Groups were compared for baseline differences prior to conducting further analyses. Chi-square tests were used to compare group differences on the demographic measures of gender, racial category, handedness, education level, current arousal level, and the type of town/city where participants had lived most of their life, with no significant differences (all *p*s > 0.20). Cognitive task performance on ANT, FFOV, and digit span measures were compared at pre-test using One-Way ANOVAs, and there were no differences between groups (all *p*s > 0.35). Additionally, One-Way ANOVAs indicated that pre-test affect (positive and negative) was similar between groups (all *p*s > 0.08).

### Image and sound ratings

Participants in the control condition were not exposed to any sounds or images, so there were no rating scores for that group. For ratings, ANOVAs were conducted comparing the conditions with sound to each other, and the conditions with pictures to each other. All image ratings are displayed in Table 1.

**Table 1:** **Ratings of relaxation for sounds and for images**.

**Rating type**	**Nature sound**	**Urban sound**	**Nature pictures**	**Urban pictures**	**Nature both**	**Urban both**	**Total**
Sound	4.75 (1.76)	2.25 (1.24)			5.43 (1.45)	2.81 (1.89)	3.82 (2.05)^c^
Image			4.50 (1.09)	3.34 (1.09)	5.13 (1.09)	3.15 (1.19)	4.05 (1.37)^c^

Only four conditions contained images, and four contained sound. Ratings were between 1 and 7, where 1 is the least relaxing and 7 is the most relaxing. Standard deviation is provided in parentheses. There was a main effect of rating for both sound and image ANOVAs.

c p < 0.001.

There was a main effect of image type, *F*_(3, 105)_ = 19.12, *p* < 0.001, *partial* η^2^ = 0.26, *p*_BIC_(H_1_ | D) = 1.00, with LSD *post-hoc* tests indicating that Nature Both was rated as the most relaxing, followed by Nature Pictures, with both significantly better than both Urban Pictures and Urban Both (all *p*s < 0.05). The urban image ratings were not significantly different from each other. Additionally, there was a main effect of sound type, *F*_(3, 107)_ = 25.74, *p* < 0.001, *partial* η^2^ = 0.30, *p*_BIC_(H_1_ | D) = 1.00. For sound, both Nature Sounds and Nature Both were rated as significantly more relaxing than both Urban groups; however, Nature Sound and Nature Both were not significantly different than each other, nor were Urban Sounds and Urban Both. This seems to indicate that participants found the nature conditions more relaxing than the urban conditions.

### Mood ratings

We examined both positive and negative affect. Scores for both can be found in Table 2.

**Table 2:** **Positive and negative affect scores on the PANAS by condition**.

	**Control**	**Nature sounds**	**Urban sounds**	**Nature pictures**	**Urban pictures**	**Nature both**	**Urban both**	**Total**
**POSITIVE AFFECT**								
Pre-test	32.7 (6.66)	34.4 (7.23)	32.6 (5.76)	33.8 (8.87)	31.2 (7.05)	33.2 (6.17)	28.9 (7.00)	32.4 (7.08)
Post-test	25.4 (8.11)	29.0 (8.45)	27.3 (7.62)	28.0 (9.74)	28.4 (7.42)	29.6 (5.93)	24.7 (7.72)	27.4 (7.96)
Difference	−7.3	−5.4	−5.3	−5.8	−2.8	−3.6	−4.2	−4.9^c^
**NEGATIVE AFFECT**								
Pre-test	14.2 (4.71)	19.2 (6.68)	16.0 (7.06)	17.8 (6.28)	16.4 (5.32)	18.8 (7.78)	15.6 (6.09)	16.8 (6.45)
Post-test	15.3 (7.21)	19.3 (9.25)	17.4 (7.74)	19.0 (8.01)	17.9 (6.73)	19.9 (8.12)	16.5 (7.58)	17.9 (7.83)
Difference	1.1	0.1	1.4	1.2	1.5	1.1	0.9	1.0^a^

Positive affect and negative affect scores shown separately. Scores range between 5 and 50, with 5 indicating a low affect in the positive/negative direction, and 50 indicating high affect. Standard deviation is provided in parentheses. There was a main effect of time for both sound and image. However, there were no main effects of condition and there was no interaction.

a p < 0.05, and

c p < 0.001.

#### Positive affect

There was a main effect of time, *F*_(1, 167)_ = 90.85, *p* < 0.001, *partial* η^2^ = 0.352, *p*_BIC_(H_1_ | D) = 1.00, with the post-test affect score (*M* = 27.5, *SD* = 7.93) dropping below the pre-test score (*M* = 32.4, *SD* = 7.00), indicating a decrease in positive affect. There was no main effect of condition, *F*_(6, 167)_ = 1.56, *p* = 0.16, *partial* η^2^ = 0.053, *p*_BIC_(H_1_ | D) = 0.00, and the interaction was not significant, *F*_(6, 167)_ = 1.20, *p* = 0.31, *partial* η^2^ = 0.041, *p*_BIC_(H_1_ | D) = 0.00, which seems to indicate that participants may have become fatigued by the experiment regardless of condition and this fatigue was not mitigated by any particular environment.

#### Negative affect

There was a main effect of time, *F*_(1, 166)_ = 5.55, *p* = 0.02, *partial* η^2^ = 0.032, *p*_BIC_(H_1_ | D) = 0.57, with the post-test affect score (*M* = 18.0, *SD* = 7.87) significantly higher than the pre-test score (*M* = 16.9, *SD* = 6.44), indicating greater levels of negative affect after completing the experiment. There was no main effect of condition, *F*_(6, 166)_ = 1.71, *p* = 0.12, *partial* η^2^ = 0.058, *p*_BIC_(H_1_ | D) = 0.00, and the interaction was not significant, *F*_(6, 167)_ = 0.17, *p* = 0.99, *partial* η^2^ = 0.006, *p*_BIC_(H_1_ | D) = 0.00, further supporting the idea that participants may have been experiencing fatigue.

### Cognitive task performance

#### Backward digit span

Backward digit span capacities as a function of experimental group are shown in Table 3. There was a main effect of time, *F*_(1, 175)_ = 5.10, *p* = 0.025, *partial* η^2^ = 0.028, *p*_BIC_(H_1_ | D) = 0.503, indicating that participants could remember more digits on the post-test (*M* = 5.66, *SD* = 1.26) than on the pre-test (*M* = 5.41, *SD* = 1.18). There was no main effect of condition, *F*_(6, 175)_ = 1.44, *p* = 0.20, *partial* η^2^ = 0.047, *p*_BIC_(H_1_ | D) = 0.00, and no significant interaction, *F*_(6, 175)_ = 1.23, *p* = 0.29, *partial* η^2^ = 0.040, *p*_BIC_(H_1_ | D) = 0.00. While participants did show some improvement in digit span capacity at post-test, those improvements were independent of the type of environment experienced, suggesting they were due to increased familiarity with the task when performing it for a second time.

**Table 3:** **Digit span capacity by condition**.

**Time**	**Control**	**Nature sounds**	**Urban sounds**	**Nature pictures**	**Urban pictures**	**Nature both**	**Urban both**	**Total**
Pre-test	4.96 (1.14)	5.30 (1.44)	5.75 (1.04)	5.28 (0.98)	5.64 (1.22)	5.40 (1.16)	5.52 (1.16)	5.41 (1.18)
Post-test	5.24 (1.09)	6.11 (1.42)	5.79 (1.23)	5.52 (1.33)	5.60 (1.23)	5.88 (1.20)	5.44 (1.25)	5.66 (1.26)
Difference	0.28	0.81	0.04	0.24	−0.04	0.48	−0.08	0.25^a^

Maximum capacity scores are 9, with minimum scores of 3. Standard deviations shown in parentheses. There was a main effect of time. However, there were no main effects of condition and there was no interaction.

a p < 0.05.

#### Attention network task

For the ANT, we only tested for differences in the executive control component, as the alerting and orienting components only require involuntary attention. Based on previous findings (Berman et al., 2008, 2012), we predicted that performance would be better for the nature conditions compared to the urban and control conditions. Additionally, we expected that the Nature Both condition would have the lowest executive control cost from pre to post-test, as it should be the most immersive. Note that better performance is indicated by a lower reaction time cost, as this is the time required to filter out incongruent information.

The means and standard deviations for each condition are reported in Table 4. There was no main effect of time, *F*_(1, 179)_ = 1.37, *p* = 0.24, *partial* η^2^ = 0.008, *p*_BIC_(H_1_ | D) = 0.13, or condition, *F*_(6, 179)_ = 0.95, *p* = 0.46, *partial* η^2^ = 0.031, *p*_BIC_(H_1_ | D) = 0.00. Additionally, the interaction of time and condition was not significant, *F*_(6, 179)_ = 0.48, *p* = 0.82, *partial* η^2^ = 0.016, *p*_BIC_(H_1_ | D) = 0.00. Contrary to previous findings, these results indicate that natural pictures and sounds did not produce any restorative effects, as indicated by more positive changes in executive function in natural relative to urban pictures and sounds.

**Table 4:** **Scores from the Attention Network Task (ANT) executive component, for all conditions**.

**Executive cost**	**Control**	**Nature sounds**	**Urban sounds**	**Nature pictures**	**Urban pictures**	**Nature both**	**Urban both**	**Total**
Pre-test	123 (62.1)	105 (36.0)	118 (36.8)	110 (52.6)	113 (32.9)	117 (54.7)	114 (41.5)	114 (46.0)
Post-test	124 (46.4)	102 (35.7)	107 (35.5)	101 (52.2)	106 (34.7)	124 (49.9)	110 (30.4)	111 (41.9)
Difference	1	−3	−11	−9	−7	7	−4	−3

Scores are in milliseconds, and represent the time difference between congruent and incongruent trials. Higher scores indicate worse performance. There were no main effects of time, condition, or any interactions.

#### Functional field of view

We also conducted Two-Way mixed ANOVAs on the FFOV reaction time scores and accuracy (separately) to determine if exposure to the different environments produced a differential improvement in performance. In this case, improvement would be indicated by a reduction in reaction time or increase in accuracy. Separate ANOVAs were conducted for 10, 20, and 30°. For all accuracy and reaction time results, refer to Table 5.

**Table 5:** **Reaction time and accuracy for all conditions on the functional field of view task**.

	**Control**	**Nature sounds**	**Urban sounds**	**Nature pictures**	**Urban pictures**	**Nature both**	**Urban both**	**Total**
**REACTION TIME—10°**								
Pre-test	949 (337)	935 (253)	951 (240)	933 (250)	993 (299)	961 (304)	909 (278)	947 (279)
Post-test	811 (268)	811 (232)	779 (204)	739 (200)	823 (246)	817 (288)	740 (200)	789 (234)
Difference	−138	−124	−172	−194	−170	−144	−169	−158^c^
**REACTION TIME—20°**								
Pre-test	949 (302)	1018 (294)	941 (224)	959 (269)	949 (292)	979 (300)	914 (263)	958 (275)
Post-test	786 (230)	834 (236)	790 (208)	743 (210)	804 (229)	816 (279)	756 (219)	790 (229)
Difference	−163	−184	−151	−216	−145	−163	−158	−168^c^
**REACTION TIME—30°**								
Pre-test	1007 (356)	1064 (381)	989 (249)	989 (263)	999 (297)	983 (317)	934 (274)	996 (306)
Post-test	836 (249)	851 (258)	804 (210)	795 (237)	834 (252)	820 (308)	767 (249)	816 (249)
Difference	−171	−213	−185	−194	−165	−163	−167	−180^c^
**ACCURACY—10°**								
Pre-test	0.839 (0.216)	0.768 (0.309)	0.791 (0.204)	0.768 (0.232)	0.729 (0.316)	0.746 (0.320)	0.828 (0.231)	0.781 (0.263)
Post-test	0.877 (0.206)	0.848 (0.151)	0.912 (0.113)	0.864 (0.151)	0.814 (0.249)	0.846 (0.266)	0.896 (0.126)	0.870 (0.203)
Difference	0.038	0.080	0.121	0.096	0.085	0.100	0.068	0.089^c^
**ACCURACY—20°**								
Pre-test	0.730 (0.280)	0.651 (0.283)	0.703 (0.243)	0.681 (0.293)	0.659 (0.321)	0.698 (0.311)	0.800 (0.225)	0.703 (0.280)
Post-test	0.814 (0.246)	0.759 (0.277)	0.849 (0.144)	0.824 (0.171)	0.754 (0.284)	0.800 (0.275)	0.861 (0.148)	0.808 (0.228)
Difference	0.084	0.108	0.146	0.143	0.095	0.102	0.061	0.105^c^
**ACCURACY—30°**								
Pre-test	0.552 (0.266)	0.543 (0.238)	0.535 (0.217)	0.511 (0.268)	0.524 (0.268)	0.564 (0.306)	0.659 (0.149)	0.554 (0.249)
Post-test	0.625 (0.264)	0.662 (0.200)	0.627 (0.174)	0.619 (0.247)	0.608 (0.256)	0.634 (0.284)	0.710 (0.147)	0.640 (0.228)
Difference	0.073	0.119	0.092	0.108	0.084	0.070	0.051	0.086^c^

Reaction times are in milliseconds, and indicate the time it took to identify the location of the target on correct trials only. An increase in accuracy from pre-test to post-test indicates an improvement in performance. For all eccentricities, there were main effects for accuracy and reaction time. However, there were no main effects of condition and no interactions.

c p < 0.001.

For all visual distances, there was a main effect of time on reaction time [10° *F*_(1, 175)_ = 126.90, *p* < 0.001, *partial* η^2^ = 0.42, *p*_BIC_(H_1_ | D) = 1.00; 20° *F*_(1, 176)_ = 197.16, *p* < 0.001, *partial* η^2^ = 0.528, *p*_BIC_(H_1_ | D) = 1.00; 30° *F*_(1, 177)_ = 164.35, *p* < 0.001, *partial* η^2^ = 0.481, *p*_BIC_(H_1_ | D) = 1.00], with a decrease in reaction time from pre- to post-test. However, there were no main effects of condition on reaction time [10° *F*_(6, 175)_ = 0.37, *p* = 0.90, *partial* η^2^ = 0.013, *p*_BIC_(H_1_ | D) = 0.00; 20° *F*_(6, 176)_ = 0.40, *p* = 0.88, *partial* η^2^ = 0.013, *p*_BIC_(H_1_ | D) = 0.00; 30° *F*_(6, 177)_ = 0.39, *p* = 0.88, *partial* η^2^ = 0.013, *p*_BIC_(H_1_ | D) = 0.00]. Additionally, none of the interactions of condition and time were significant [10° *F*_(6, 175)_ = 0.41, *p* = 0.87, *partial* η^2^ = 0.014, *p*_BIC_(H_1_ | D) = 0.00; 20° *F*_(6, 176)_ = 0.58, *p* = 0.75, *partial* η^2^ = 0.019, *p*_BIC_(H_1_ | D) = 0.00; 30° *F*_(6, 177)_ = 0.26, *p* = 0.95, *partial* η^2^ = 0.009, *p*_BIC_(H_1_ | D) = 0.00]. While these data consistently indicate that participants were getting better at the task over time, there was no indication of environmental condition producing a difference in restorative effects that influenced performance on this cognitive task, and overall, the data are better explained as practice effects.

The same pattern of improvement was observed for accuracy, with significant main effects of time on accuracy [10° *F*_(1, 170)_ = 59.66, *p* < 0.001, *partial* η^2^ = 0.26, *p*_BIC_(H_1_ | D) = 1.00; 20° *F*_(1, 174)_ = 85.16, *p* < 0.001, *partial* η^2^ = 0.329, *p*_BIC_(H_1_ | D) = 1.00; 30° *F*_(1, 170)_ = 59.32, *p* < 0.001, *partial* η^2^ = 0.259, *p*_BIC_(H_1_ | D) = 1.00]. Once again, there was no main effect of condition on accuracy [10° *F*_(6, 170)_ = 0.80, *p* = 0.80, *partial* η^2^ = 0.018, *p*_BIC_(H_1_ | D) = 0.00; 20° *F*_(6, 174)_ = 0.82, *p* = 0.56, *partial* η^2^ = 0.027, *p*_BIC_(H_1_ | D) = 0.00; 30° *F*_(6, 170)_ = 0.75, *p* = 0.61, *partial* η^2^ = 0.026, *p*_BIC_(H_1_ | D) = 0.00], nor were there any interactions of condition and time [10° *F*_(6, 170)_ = 0.90, *p* = 0.50, *partial* η^2^ = 0.031, *p*_BIC_(H_1_ | D) = 0.00; 20° *F*_(6, 174)_ = 1.00, *p* = 0.43, *partial* η^2^ = 0.033, *p*_BIC_(H_1_ | D) = 0.00; and 30° *F*_(6, 170)_ = 0.60, *p* = 0.73, *partial* η^2^ = 0.021, *p*_BIC_(H_1_ | D) = 0.00].

In summary, our findings suggest that participants become more accurate and faster at the FFOV task, indicating a practice effect. However, there were no effects of the experimental conditions.

## Discussion

Based on previous research on ART (Tennessen and Cimprich, 1995; Hartig et al., 2003; Berman et al., 2008, 2012; Taylor and Kuo, 2009), we predicted that images and sounds of natural environments would have a restorative effect on direct attention, thus resulting in improved performance on cognitive tasks that require sustained direct attention. However, in all three of our cognitive tasks we failed to find support for this hypothesis, a finding inconsistent with some of the previous research in the cognitive restoration domain (e.g., Hartig et al., 2003; Berman et al., 2008), but consistent with other studies that have failed to replicate restorative effects. For example, while Hartig et al. (2003) found an improvement in performance on the necker cube task when participants were exposed to natural settings, they were unable to replicate previous findings (Hartig et al., 1996) non search and memory tests.

Consistent with prior studies (Hartig and Staats, 2006; White et al., 2010), our prediction that nature sounds and images would be percieved as more relaxing than urban sounds and images was supported. However, while previous SRT research would predict a restorative effect would be reflected in affect (Ulrich, 1983; Hartig et al., 2003; van den Berg et al., 2003), we were surprised to see that our participants scored significantly higher on the negative affect component of the PANAS, and significantly lower on the positive affect component at post-test compared to pre-test. Interestingly, these effects were consistent across all restoration conditions. This may suggest that the cognitive tasks resulted in some fatigue, and that the restoration periods (regardless of the type) were not sufficient to overcome the fatigue. Previous research would lead us to believe that the restorative effects of the nature conditions should help overcome the effects of fatigue on the cognitive tasks, and that it should do the same for affect, so it is surprising that this was not the case in our study. Alternatively, it is also possible that the tasks we used in and of themselves may have contributed to the general trend away from positive and toward negative affect over time. Consider, for example, that sitting on a real beach does not require much thought, nor does it require any real-world analog to our rating task. Hence, the rating task that participants were required to perform may have unintendidly irritated participants, though it should be noted that we have no evidence of this. Similarly, viewing many highly similar nature pictures may have induced boredom, and this in turn may have also contributed to the trend toward higher negative affect over time. Again, we have no direct evidence, however, that this was in fact the case.

Notably, there was an absence of any restorative effect on the executive component of the ANT. In previous work (Berman et al., 2008), the effect of a restoration period associated with natural environments on this measure was particularly notable. In our study, we found no evidence of restorative effects on executive function as measured by the ANT task. It is possible that there were some subtle methodological differences between our study and previous work that may have contributed to these inconsistent findings, possibly including our use of acquatic environments. However, since blue spaces containing vegetation have been reported to be perceived as the most restorative (White et al., 2010), we do not believe this would result in the lack of an effect in our study. Overall, our data appear to be quite consistent and offer support the for the possibility that restorative stimuli, viewed or listened to in the lab, may not be sufficient for producing acute improvements in exective function, at least in the context measured here.

In addition to characterizing previously studied contexts within which cognitive restoration has been shown to occur, our study is also the first, to our knowledge, to explore whether the FFOV task is amenable to cognitive restoration. Unfortunately, our data provided no evidence that attentional breadth, as measured by the FFOV, is sensitive to a cognitive restoration period, when presented on a computer screen or auditorily through a headset. Perhaps exposure to more immersive restoration techniques, such as an actual walk through a natural environment, might produce a different outcome. Given that the FFOV has been shown to have strong relationships to tasks requiring directed attention, such as driving (e.g., Crundall et al., 1999; Roenker et al., 2003; Atchley and Dressel, 2004), further examination of the task might prove fruitful.

Our study is also, to our knowledge, the first in this domain to exclusively look at ocean images as opposed to other natural environments, which are generally composed of greenery. This decision was partly influenced by previous work indicating that the presence of blue space may be particularly robust in inducing the cognitive restoration effect (White et al., 2010). Using ocean-based images also allowed us a level of control over the nature image set that has been somewhat elusive in previous studies. Given previous findings, we expected that ocean images would evoke a greater level of restoration than park-like images; however, our data did not support our prediction. In fact, our findings strongly suggest that the effect of the color blue in natural environments may not be as restorative as previously suggested.

There are a number factors that may have contributed to our failure to replicate some previous findings of restoration effects on cognition, despite our having used a fairly typical paradigm from the literature. One possibility is that a bias against the publication of null effects might be preventing some failures to replicate from reaching the broader community. Another possibility, which has not received much attention, is that performance on the cognitive tasks that we (as well as others) used could be moderated by arousal levels (Yerkes and Dodson, 1908; Solomon and Corbit, 1974; Thompson et al., 2001). One adaptation of the popular Yerkes-Dodson law can be instantiated though an inverted-U curve describing the relationship between performance and arousal. When arousal is too low or too high, performance suffers; when it is moderate, performance is at its peak. From this, it is possible that our nature condition reduced arousal to such a level that the relationship between restoration and performance was moderated by arousal, reducing performance from what may have occurred had arousal been moderate. Conversely, the arousal level for the urban conditions may have resulted in an increased level of performance. The combination of these two may have diluted the effect, thus resulting in a null result. It is also possible that the rating task itself inhibited the restorative effect by causing additional fatigue, though this was likely insufficient to cause the null result, as previous studies (Berman et al., 2008) have used similar tasks. Another possibility is that the overall task may not have sufficiently caused fatigue, since the control condition also exhibited similar practice effects as the other groups.

Another potential explanation could be the duration of the restoration period. Many of the experiments that find restorative effects take place with walks lasting nearly an hour. Although effects have been previously observed in studies using the same restoration period duration that we used (Berman et al., 2008), other studies suggest that performance differences may not appear when restoration exposure remains less than 15–20 min (Hartig et al., 1996, 2003; Laumann et al., 2003). Additionally, it is possible that the presence of people in our urban images could could have interfered with the restorative effects. Although little of the previous research has attempted to control for the presence of people, the presence of others has been shown to effect the perception of restorativeness of different types of environments (Staats and Hartig, 2004).

## Conclusion

ART has garnered a good deal of support since it was first proposed. In particular, its relationship with cognitive performance has been studied with various tasks, ranging from walking through real-world environments, to viewing simple sets of images. While ART and SRT indicate that the restorative effects of nature should be observed after a restoration period, we were unable to find evidence in support of these assertions in a variety of cognitive tasks, despite designing our restoration period to provide all four requirements of restorative environments. Instead, our data suggest that short term exposure to images and sounds of nature do not provide any additional cognitive benefit above exposure to urban environments. Importantly, our null results are supported not only by traditional ANOVA, but also by bayesion posterior probabilities. Further investigation should be conducted to determine where cognitive restoration occurs and under what conditions, and should also investigate other cognitive tasks that may help build a better understanding of how this restoration can have real-world implications.

### Conflict of interest statement

The authors declare that the research was conducted in the absence of any commercial or financial relationships that could be construed as a potential conflict of interest.

## References

[B1] Atchley P. Dressel J. (2004). Conversation limits the functional field of view. Hum. Factors 46, 664–673 10.1518/hfes.46.4.664.56808 15709328

[B2] Ball K. Edwards J. D. Ross L. A. (2007). The impact of speed of processing training on cognitive and everyday functions. J. Gerontol. B Psychol. Sci. Soc. Sci. 62, 19–31 10.1093/geronb/62.special_issue_1.19 17565162

[B3] Ball K. K. Beard B. L. Roenker D. L. Miller R. L. Griggs D. S. (1988). Age and visual search: expanding the useful field of view. J. Opt. Soc. Am. A 5, 2210–2219 10.1364/JOSAA.5.002210 3230491

[B4] Belchior P. Marsiske M. Sisco S. M. Yam A. Bavelier D. Ball K. (2013). Video game training to improve selective visual attention in older adults. Comput. Human Behav. 29, 1318–1324 10.1016/j.chb.2013.01.034 24003265 PMC3758751

[B5] Berman M. G. Jonides J. Kaplan S. (2008). The cognitive benefits of interacting with nature. Psychol. Sci. 19, 1207–1212 10.1111/j.1467-9280.2008.02225.x 19121124

[B6] Berman M. G. Kross E. Krpan K. M. Askren M. K. Burson A. Deldin P. J. (2012). Interacting with nature improves cognition and affect for individuals with depression. J. Affect. Disord. 140, 300–305 10.1016/j.jad.2012.03.012 22464936 PMC3393816

[B7] Bouma H. (1978). Visual search and reading: eye movements and functional visual field: a tutorial review, in Attention and Performance VII, ed Requin J. (Hillsdale, NJ: Erlbaum), 115–146

[B8] Bowler D. Buyung-Ali L. Knight T. Pullin A. (2010). A systematic review of evidence for the added benefits to health of exposure to natural environments. BMC Public Health 10:456 10.1186/1471-2458-10-456 20684754 PMC2924288

[B9] Brown E. Cairns P. (2004). A grounded investigation of game immersion, in CHI '04 Extended Abstracts on Human Factors in Computing Systems (New York, NY: ACM), 1297–1300

[B10] Cowan N. (2001). The magical number 4 in short-term memory: a reconsideration of mental storage capacity. Behav. Brain Sci. 24, 87–114 10.1017/S0140525X01003922 11515286

[B11] Crundall D. Underwood G. Chapman P. (1999). Driving experience and the functional field of view. Perception 28, 1075–1088 10.1068/p2894 10694958

[B12] Depledge M. H. Stone R. J. Bird W. J. (2011). Can natural and virtual environments be used to promote improved human health and wellbeing? Environ. Sci. Technol. 45, 4660–4665 10.1021/es103907m 21504154

[B13] Engel F. L. (1971). Visual conspicuity, directed attention and retinal locus. Vision Res. 11, 563–575 10.1016/0042-6989(71)90077-0 5558576

[B14] Engel F. L. (1977). Visual conspicuity, visual search and fixation tendencies of the eye. Vision Res. 17, 95–108 10.1016/0042-6989(77)90207-3 855214

[B15] Eriksen B. A. Eriksen C. W. (1971). Effects of noise letters upon the identification of a target letter in a nonsearch task. Percept. Psychophys. 16, 143–149 10.3758/BF03203267

[B16] Fan J. McCandliss B. D. Fossella J. Flombaum J. I. Posner M. I. (2005). The activation of attentional networks. Neuroimage 26, 471–479 10.1016/j.neuroimage.2005.02.004 15907304

[B17] Fan J. McCandliss B. D. Sommer T. Raz A. Posner M. I. (2002). Testing the efficiency and independence of attentional networks. J. Cogn. Neurosci. 14, 340–347 10.1162/089892902317361886 11970796

[B18] Grimshaw M. (2008). Sound and immersion in the first-person shooter. Int. J. Intell. Games Simul. 5, 119–124

[B19] Grimshaw M. Lindley C. A. Nacke L. (2008). Sound and immersion in the first-person shooter: mixed measurement of the player's sonic experience, in Proceedings of Audio Mostly Conference (Piteå), 1–7

[B19a] Hartig T. Böök A. Garvill J. Olsson T. Gärling T. (1996). Environmental influences on psychological restoration. Scand. J. Psychol. 37, 378–393 10.1111/j.1467-9450.1996.tb00670.x 8931393

[B20] Hartig T. Evans G. W. Jamner L. D. Davis D. S. Garling T. (2003). Tracking restoration in natural and urban field settings. J. Environ. Psychol. 23, 109–123 10.1016/S0272-4944(02)00109-3

[B21] Hartig T. Staats H. (2006). The need for psychological restoration as a determinant of environmental preferences. J. Environ. Psychol. 26, 215–226 10.1016/J.JENVP.2006.07.007

[B22] Herzog T. R. Black A. M. Fountaine K. A. Knotts D. J. (1997). Reflection and attentional recovery as distinctive benefits of restorative environments. J. Environ. Psychol. 17, 165–170 10.1006/jevp.1997.0051

[B23] Hoddes E. Zarcone V. Smythe H. Phillips R. Dement W. C. (1973). Quantification of sleepiness: a new approach. Psychophysiology 10, 431–436 4719486 10.1111/j.1469-8986.1973.tb00801.x

[B24] Joseph B. (Composer) (2010). Ocean Waves. On Ocean Waves [CD]. Vadnals Heights; Minnesota: Robbins Island Music

[B25] Kaplan R. Kaplan S. (1989). The Experience of Nature: A Psychological Perspective. Cambridge: Cambridge University Press

[B26] Kaplan S. (1995). The restorative benefits of nature: toward an integrative framework. J. Environ. Psychol. 15, 169–182 10.1016/0272-4944(95)90001-2

[B27] Kaplan S. Bardwell L. B. Slakter D. B. (1993). The museum as a restorative environment. Environ. Behav. 25, 725–742 10.1177/001391659325600424639076

[B28] Larsen V. A. (2011). When the Going Gets Tough, Will Nature Get you Going? The Effect of Water, Natural and Urban Landscapes on Cognitive Control. Unpublished doctoral dissertation, University of Oslo, Oslo

[B29] Laumann K. Gärling T. Stormark K. M. (2003). Selective attention and heart rate responses to natural and urban environments. J. Environ. Psychol. 23, 125–134 10.1016/S0272-4944(02)00110-X

[B30] Lechtzin N. Busse A. M. Smith M. T. Grossman S. Nesbit S. Diette G. B. (2010). A randomized trial of nature scenery and sounds versus urban scenery and sounds to reduce pain in adults undergoing bone marrow aspirate and biopsy. J. Alter. Complement. Med. 16, 965–972 10.1089/acm.2009.0531 20799901 PMC3110836

[B31] Mackworth N. H. (1965). Visual noise causes tunnel vision. Psychon. Sci. 3, 67–68 21842405

[B32] Masson M. E. (2011). A tutorial on practical Bayesian alternative to null-hypothesis significance testing. Behav. Res. Methods 43, 679–690 10.3758/s13428-010-0049-5 21302025

[B33] Olmsted F. L. (1870). Public Parks and the Englargement of Towns. Cambridge, MA: Riverside Press

[B34] Posner M. I. (1980). Orienting of attention. Q. J. Exp. Psychol. 32, 3–25 10.1080/00335558008248231 7367577

[B35] Ratcliffe E. Gatersleben B. Sowden P. T. (2013). Bird sounds and their contributions to perceived attention restoration and stress recovery. J. Environ. Psychol. 36, 221–228 10.1016/j.jenvp.2013.08.004

[B37] Roenker D. L. Cissell G. M. Ball K. K. Wadley V. G. Edwards J. D. (2003). Speed-of-processing and driving simulator training result in improved driving performance. Hum. Factors 45, 218–233 10.1518/hfes.45.2.218.27241 14529195

[B38] Sanders T. Cairns P. (2010). Time perception, immersion and music in videogames, in Proceedings of the 24th BCS Interaction Specialist Group Conference (Swinton), 160–167

[B39] Serafin S. Serafin G. (2004). Sound design to enhance presence in photorealistic virtual reality, in Proceedings of ICAD 04-Tenth Meeting of the International Conference on Auditory Display (Sydney, NSW).

[B40] Solomon R. L. Corbit J. D. (1974). An opponent-process theory of motivation. I. Temporal dynamics of affect. Psychol. Rev. 81, 119–145 10.1037/h0036128 4817611

[B41] Staats H. Hartig T. (2004). Alone or with a friend: a social context for psychological restoration and environmental preferences. J. Environ. Psychol. 24, 199–211 10.1016/j.jenvp.2003.12.005

[B42] Stone R. J. (2008). Human Factors Guidelines for Interatcive 3D and Game-Based Training Systems Design, 1st Edn. Human Factors Integration Defence Technology Centre Publication. Available online at: http://www.hfidtc.com

[B43] Taylor A. F. Kuo F. E. (2009). Children with attention deficits concentrate better after walk in the park. J. Affect. Disord. 12, 402–409 10.1177/1087054708323000 18725656

[B44] Taylor A. F. Kuo F. E. Sullivan W. C. (2002). Views of nature and self-discipline: evidence from inner city children. J. Environ. Psychol. 22, 49–63 10.1006/jevp.2001.0241

[B45] Tennessen C. M. Cimprich B. (1995). Views to nature: effects on attention. J. Environ. Psychol. 15, 77–85 10.1016/0272-4944(95)90016-0

[B46] Thompson W. F. Schellenberg E. G. Husain G. (2001). Arousal, mood, and the mozart effect. Psychol. Sci. 12, 248–251 10.1111/1467-9280.00345 11437309

[B47] Times Square (2000). On The Sound of New York City [CD]. LoganMedia.

[B48] Ulrich R. S. (1983). Aesthetic and affective response to natural environment, in Behavior and the Natural Environment, eds Altman I. Wohlwill J. F. (New Yrok, NY: Plenum Press), 85–125

[B49] Ulrich R. S. Simons R. F. Losito B. D. Fiorito E. Miles M. A. Zelson M. (1991). Stress recovery during exposure to natural and urban environments. J. Environ. Psychol. 11, 201–230 10.1016/S0272-4944(05)80184-720617017

[B50] Urbanization (2011). U.S. Central Intelligence Agency. From CIA World Factbook. Available online at: https://www.cia.gov/library/publications/the-world-factbook/fields/2212.html (Retrieved June 17, 2013).

[B50a] van den Berg A. E. Custers M. H. G. (2011). Gardening promotes neuroendocrine and affective restoration from stress. J. Health Psychol. 16, 3–11 10.1177/1359105310365577 20522508

[B51] van den Berg A. E. Koole S. L. Van der Wulp N. Y. (2003). Environmental preference and restoration: (How) are they related? J. Environ. Psychol. 23, 135–146 10.1016/S0272-4944(02)00111-1

[B52] Velarde M. D. Fry G. Tveit M. (2007). Health effects of viewing landscapes -landscape types in environmental psychology. Urban For. Urban Gree. 6, 199–212 10.1016/j.ufug.2007.07.001

[B53] Watson D. Clark L. A. (1988). Development and validation of breif measures of positive and negative affect: the PANAS scales. J. Pers. Soc. Psychol. 54, 1063–1070 10.1037/0022-3514.54.6.1063 3397865

[B54] White M. Smith A. Humphryes K. Pahl S. Snelling D. Depledge M. (2010). Blue space: the importance of water for preference, affect, and restorativeness ratings of natural and built scenes. J. Environ. Psychol. 30, 482–493 10.1016/j.jenvp.2010.04.004

[B55] White M. P. Pahl S. Ashbullby K. Herbert S. Depledge M. H. (2013). Feelings of restoration from recent nature visits. J. Environ. Psychol. 35, 40–51 10.1016/j.jenvp.2013.04.002

[B56] Yerkes R. M. Dodson J. D. (1908). The relation of strength of stimulus to rapidity of habit-forming. J. Comp. Neurol. Psychol. 18, 459–482 10.1002/cne.920180503

